# Seeds of Success: Empowering Latina STEM Girl Ambassadors Through Role Models, Leadership, and STEM-Related Experiences

**Published:** 2023-09-26

**Authors:** Liz Hernández-Matías, Greetchen Díaz-Muñoz, Giovanna Guerrero-Medina

**Affiliations:** 1 Ciencia Puerto Rico, San Juan, PR; 2 Office of Diversity, Equity and Inclusion, Yale School of Medicine, New Haven, CT

**Keywords:** Latinas, Out-of-school Programs, Role Models, STEM Experiences, Leadership, Underserved Communities, Mentors, Girls in STEM

## Abstract

Gender stereotypes and lack of access to relatable role models, mentors, and STEM opportunities have been suggested to deter middle school girls and students from underrepresented backgrounds away from STEM. Seeds of Success, an out-of-school program, is designed to inspire girls to consider STEM careers by countering gender stereotypes through relatable role models, promoting STEM confidence through STEM workshops and hands-on activities, and encouraging alignment between cultural and STEM identities through community-based STEM projects that develop leadership skills. Since 2015, the program has impacted 453 students who in turn have reached more than 42,777 people in Puerto Rico through their STEM Ambassadors projects. A robust mix-method evaluation of the 2020 and 2021 cohorts demonstrates significant improvements in participants’ STEM attitudes and science identity, as well as in their self-perception as a leader, confidence in their ability to succeed in science, knowledge about STEM careers and opportunities, and access to STEM role models. Moreover, 95% of participants intend to continue participating in STEM activities after the program and overall scores for the entire survey were significantly higher after the program than before. We discuss lessons learned for other programs seeking to empower girls from historically underserved backgrounds in STEM.

## INTRODUCTION

Science, technology, engineering, and math (STEM) careers are among the highest-paying and fastest-growing careers in the U.S. (U.S. Bureau of Labor Statistics, 2020). Yet, women and minorities are underrepresented in majors that lead to careers in these high-demand fields. According to the National Science Foundation (2017), Latinas and African American women comprised 3.4% of the 2015 STEM workforce, despite being 15% of the U.S. population. Several factors may contribute to these disparities. Although girls perform as well as boys in science and math in grade school and middle school, persistent, subconscious stereotypes of male scientists or engineers may discourage girls away from STEM disciplines (Nosek et al., 2009; Shapiro and Williams, 2012). Parental expectations and peer norms and a perceived lack of fit with personal goals or values may play a role in gender differences (Cheryan et al., 2015; Dasgupta and Stout, 2014; Starr and Leaper, 2019).

There are additional challenges with respect to girls from underrepresented or low-resource backgrounds who are more likely to lack access to STEM courses, resources, and supportive environments. For example, Hispanic high school students are interested in STEM subjects and careers at rates similar to those of students from majority groups. But Latinx high school students exhibit significantly lower STEM participation, preparation, college plans, and confidence in their STEM abilities (Modi et al., 2012; Sorge et al., 2000; Student Research Foundation, 2020). A lack of access to STEM role models, mentors, and local resources is associated with lower participation and preparation of Latinx students in STEM (Student Research Foundation, 2020).

**Figure 1.**
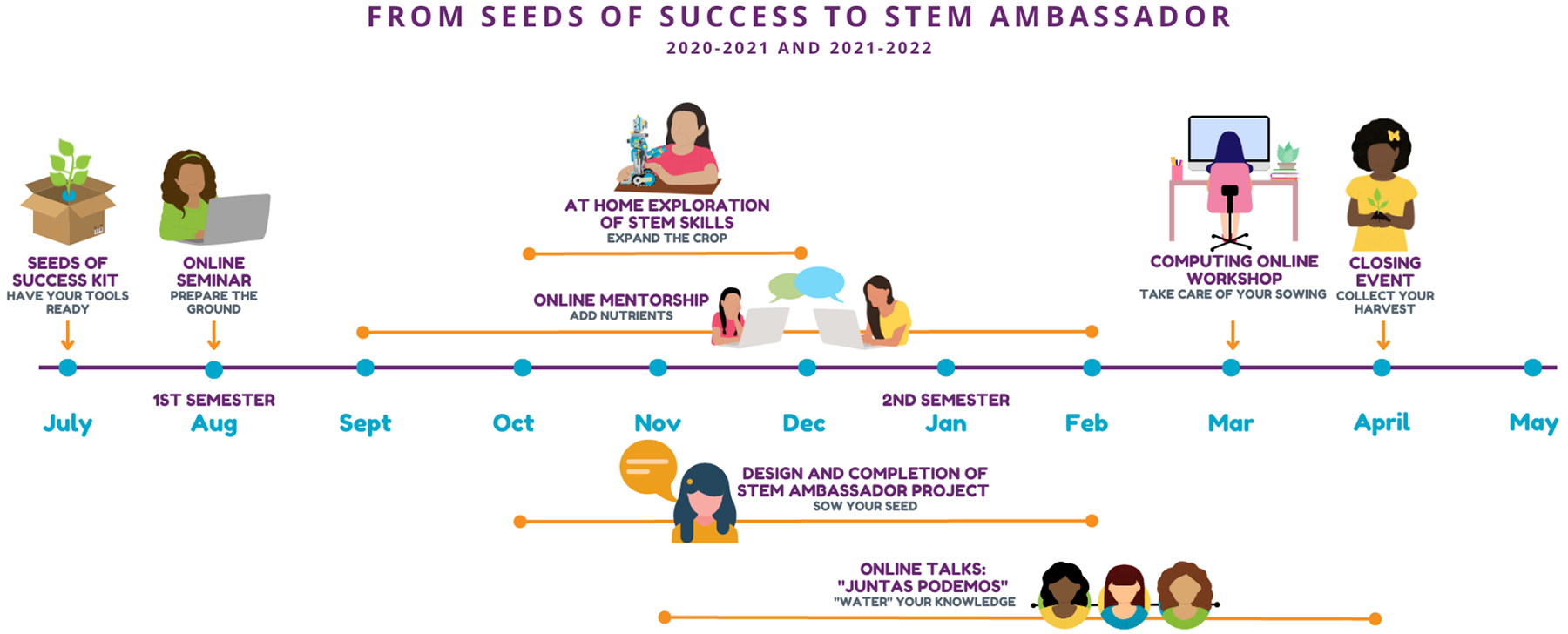
Program overview for cohorts of 2020–2021 and 2021–2022.

Three strategies have shown to be successful in countering the factors that result in gender and race disparities: experience and skill development for fostering self-efficacy, exposure to relatable role models to improve STEM attitudes and increase science identity, and connectedness to community to reinforce the ‘fit’ between cultural and STEM identities. Based on this body of evidence, we established a set of activities (Figure 1) for the Seeds of Success Girls in STEM Ambassadors Program with the following objectives: (1) expose girls to problem-solving and critical-thinking skills through STEM activities, (2) inspire girls to envision STEM careers through interactions with culturally-similar and relatable STEM role models, and (3) empower girls as members of a STEM Ambassadors group to apply leadership skills to inspire peers in their schools and their communities. Recruitment of girls and mentors, as well as the dissemination of the program and its outcomes, benefited from Ciencia Puerto Rico, a nonprofit organization that provides a virtual home for >16,000 scientists, students, educators, and allies with backgrounds or interests in Puerto Rico (PR).

### Building Skills Through STEM Experiences.

Hands-on activities increase interest, self-efficacy, and engagement in STEM and instill positive perceptions of scientists (Cleaves, 2005; Prieto-Rodriguez et al., 2020). In male-dominated fields like computer science, which is not part of the K-12 curriculum in many districts, out-of-school experiences in welcoming environments can be instrumental in providing girls from underrepresented backgrounds the skills and knowledge needed to build self-efficacy (Accenture, 2016). Within our program, each girl receives a *Seeds of Success Kit* with educational materials related to STEM and STEM careers, a Foldscope activity to complete at home (Cybulski et al. 2014) and materials for an online chemistry workshop focused on concepts such as acids, bases, pH, soil, buffers, and indicators, which are common topics in the 7th-9th grade curriculum. Girls are coached during the live, interactive webinars to complete the STEM activities and report their outcomes. Girls also participate in an online coding/robotics workshop to develop basic coding skills and to consider how technology can be applied to solve interdisciplinary problems (Figure 1).

### Relatable Role Models and Mentors.

Given the low numbers of women of color in STEM and in media portrayals of STEM professionals (National Science Foundation, 2017; Lyda Hill, 2018), girls from underrepresented or underserved backgrounds are less likely to have access to STEM role models with whom they identify. This lack of representation may preclude envisioning a STEM career. For girls and students from underrepresented backgrounds, exposure to relatable role models promotes a sense of belonging and challenges the stereotypes that perpetuate notions of unsuitability or incapacity in STEM (Prieto-Rodriguez et al., 2020). Mentoring relationships offer girls opportunities to directly challenge stereotypes and help girls grow socially and emotionally. In-group mentors increase feelings of belonging, motivation, and confidence in STEM, leading to greater STEM career aspirations and retention in STEM majors (Blake-Beard et al., 2011; Dennehy and Dasgupta, 2017). Seeds of Success addresses the lack of access to relatable STEM professionals by connecting girls to diverse female STEM role models to promote science identity and STEM career exploration. Hundreds of diverse female STEM role models are enlisted as online mentors via the CienciaPR platform, as well as through collaborations with local corporate funders (e.g., Amgen, J&J, Medtronic). Girls participate in structured small-group mentoring sessions and individual online meetings with assigned mentors. Girls also interact online with other women STEM leaders through the “Juntas Podemos” [Together We Can] webinar series (open to a broader audience) and have access to our growing digital collection of Latina Women in STEM (Figure 1).

### Aligning Self- and Science Identity Through Community-Oriented Projects.

Middle and high school students have better learning outcomes when science is presented in a context that includes personal associations and connections to culture, reality, and experiences (Christy, 2016; Vogt et al., 2016). Community-relevant projects engage students who share their community’s values, who don’t see science as part of their cultural or social identity, or who perceive that STEM careers do not offer an opportunity to connect with others in their community (Fuesting and Diekman, 2017; Masnick et al., 2009). Women and underrepresented minorities frequently cite community-oriented values as motivators for pursuing STEM careers (Gibbs and Griffin, 2013; Kozoll and Osborne, 2004; Thoman et al., 2014). Through Seed of Success, girls receive leadership training and guidance to complete a STEM Ambassador outreach project that impacts at least 30 additional people from their schools and/or communities. We partner with science and education organizations in Puerto Rico to support girls as they disseminate their projects and connect with their communities. Girls also present their projects to friends, mentors, and community members in a closing STEM Achievement Closing Ceremony (Figure 1).

We have run five cohorts of Seeds of Success with middle and high school girls since 2015. Middle school is a critical stage during which students’ STEM interest is influenced and their preparation to pursue college degrees in STEM disciplines is shaped (Maltese and Tai, 2011; Tai et. al., 2006). Since 2020, we have focused our program solely on middle school girls (7th-9th grade) as our primary target. Although the program was initially established as fully in-person, in 2020 all activities were converted to an online format to continue the program despite COVID-19. While COVID-19 presented a major challenge, it allowed us to expand the number of activities offered to girls and innovate and develop new resources such as the largest digital collection of Latina STEM professionals and a new online mentoring program. The program continued fully online in 2021 with the exception of the program’s closing ceremony. In this article, we are including data for the 2020–2021 and 2021–2022 cohorts as program design, program methods and evaluation are comparable.

## METHODS

### Design.

Yale University’s Institutional Review Board approved this study. Study participation required the signed informed consent of a parent or guardian and the child’s assent. Participants were assured anonymity and did not receive any incentive for participation. A mixed-methods design was used to understand how the experience of participating in the program impacted girls’ (1) science identity, (2) main program objectives, (3) STEM interest, (4) leadership identity, (5) STEM attitudes, and (6) numerical skills and perception. We also inquired about their satisfaction with program activities.

### Instruments.

A Pre-Intervention Survey consists of 52 closed-ended questions. Questions focus on participants’: (1) demographic characteristics, (2) science identity, (3), program objectives (4) STEM interest (5) leadership identity (6) STEM attitudes and (7) numerical skills and perception. Most questions are presented as 5-point Likert items. A few negative items are included to avoid acquiescence bias. Questions regarding participants’ perceptions of scientists come from the General Social Survey (Smith, 2019); questions related to self-efficacy and outcome expectations come from the Self-Efficacy Scale (Fouad, 1997); questions regarding science identity come from the Science Identity Survey (SISE) (Schon, 2015; Hernandez-Matias, 2019); and questions related to previous exposure to STEM, interest, and intention come from the Core STEM Survey (Marra, 2004).

A Mid-point Satisfaction Survey consists of 30 Likert questions and four open-ended questions to examine the students’ satisfaction with the program activities. Questions are based on a published survey from “Assessing Women in Engineering” (Marra, 2004).

A Post-Intervention Survey includes the Pre-Intervention non-demographic question set plus questions related to the girl’s satisfaction with the program’s components and overall impression of the program’s impact.

All instruments were sent via email to program participants and collected through the Yale Qualtrics XM online survey platform. Surveys were tracked with a randomly generated ID number assigned to each participant at the beginning of the study. The Pre-Intervention Survey was sent after program orientation, the Mid-point Satisfaction Survey 4-months into the program, and the Post- Intervention Survey at the end of the program.

A STEM Role Model Survey consists of 12 multiple selection questions, seven Likert questions, and three open-ended questions focused on (1) academic stage or professional work sector, (2) previous mentoring experience, (3) participation satisfaction and impressions, and (4) perceived impact of the program on themselves and on the girls. Surveys were sent via email at the end of the program.

### Quantitative Data.

Survey descriptive statistics, reliability, and goodness-of-fit were calculated using IBM SPSS Statistics software package, v.29. The survey items and instruments have undergone reliability and validity testing. Cronbach’s alpha was used to estimate the internal consistency of the survey using the following measurement criterion: α ≥0.90 (high), α ≥0.80 (good), α ≥0.70 (adequate) (Nunnally, 1978). The Kaiser-Meyer-Olkin (KMO) measure of sampling adequacy threshold was set at −2 to +2 and kurtosis at −7 to +7 (Hair et al., 2010; Bryne 2010). Exploratory Factor Analysis (EFA) with principal axis extraction method was selected at 95 percentile to determine interpretable constructs that explain correlations among measurable variables (Preacher and McCallum, 2003; Knekta et al., 2019; O’Connor, 2000). We rotated the factor solutions using the oblimin method, which allowed correlation among factors (Preacher and McCallum, 2003). We analyzed Likert-item responses within the questionnaires as ordinal data using the median response and used Wilcoxon Rank-Sum tests to examine significance.

### Qualitative Data.

Content analysis was used to interpret participants’ experiences expressed in the open survey questions. A deductive approach was used to study program impact. We focused on manifest content to code the text’s visible and surface content. Before beginning analysis, we identified preconceived categories from the literature. Data were coded according to the predetermined categories. The reported quotes were taken from open-ended questions.

## RESULTS

### Program Participants.

The program application was disseminated through CienciaPR’s website (~40K visitors per month), social media channels (reach of ~30K per week), and newsletter (>12,000 subscribers in 2020 and 2021), as well as through a media campaign that included a press release and radio and TV interviews with past students. School directors and teachers were also alerted about the application through a collaboration with the Department of Education of Puerto Rico (DEPR). In 2020 and 2021 we received 476 and 305 applications, respectively.

Participants were selected by a committee of past mentors and based on a rubric that prioritized (a) need (i.e., lack of prior exposure to STEM, low socioeconomic settings or public schools, or being in the last grade eligible for the program), (b) interest (i.e., clear interest in the program or history of past applications), (c) leadership potential (i.e., prior experience with community projects or concrete ideas for STEM Ambassador projects), and (d) geographic diversity (i.e., selection took into account the diversity of municipalities and schools across PR).

**Figure 2.**
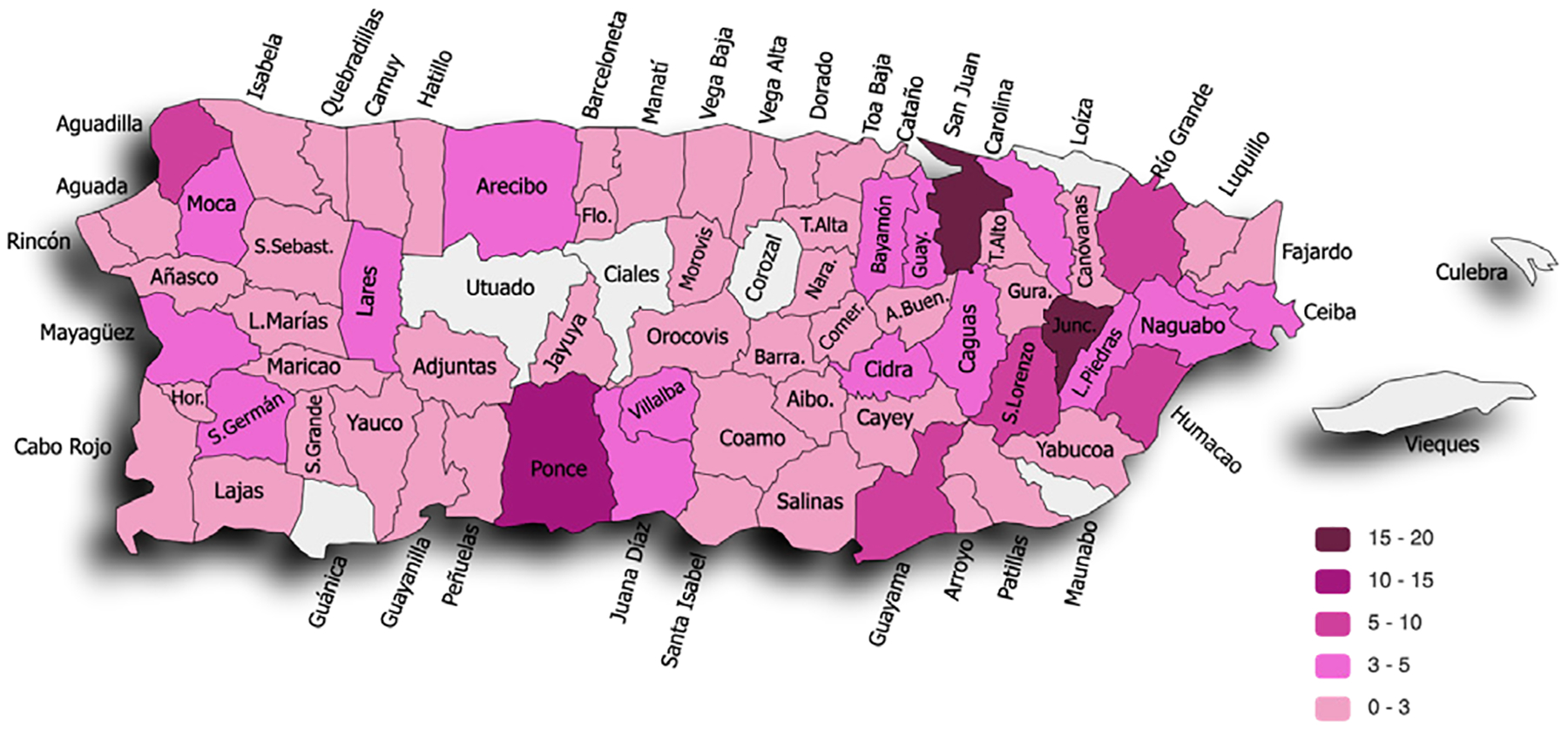
Distribution of 2020 and 2021 program participants across Puerto Rican municipalities.

A total of 241 girls participated in the program during the reported cycles (121 in 2020 and 120 in 2021). Participating girls came from 73 out of 78 municipalities in Puerto Rico (Figure 2) and included girls in 7th (18%), 8th (36%), and 9th (46%) grade. A majority of participants were from public schools (64%), with other girls coming from private schools (31%) or homeschooling (5%).

In order to receive their completion certificate and invitation to the closing ceremony, girls were required to participate (live if possible or asynchronously if they had a conflict) in the program orientation and leadership workshop, at least one mentoring session, two of the “Juntas Podemos” webinars, and a chemistry of everyday life hands-on workshop focused on concepts such as chemical reactions, pH, acids and bases, indicators and buffers, delivered by students leaders from the University of Puerto Rico, Rio Piedras’ student American Chemical Society (ACS) chapter. Although delivered online, participants were encouraged to interact with each other by commenting on each other’s questions or progress using the chat function or live in breakout groups during the peer mentoring sessions and hands-on workshops. Participants who completed these requirements and completed the community outreach project were recognized as STEM Ambassadors during the closing ceremony.

To promote engagement, program communications strategies were based on videos, WhatsApp text messages, and posts that celebrated girls’ efforts in completing the required activities. These strategies were based on a 2020 survey that indicated that parents preferred WhatsApp compared to email. We actively monitor girls’ participation and potential risk of drop-out. When a girl notifies us that she is not continuing in the program, or when we notice a number of absences or incomplete milestones, a meeting is scheduled with the parents to understand the reasons and identify alternative participation plans.

All participants both years completed the leadership workshop, 80% completed at least 1 mentoring session with a Latina STEM role model, 71% completed requirements for the final certificate, and 62% completed the final STEM Ambassadors project (Table 1).

### STEM Ambassador Projects.

**Figure 3.**
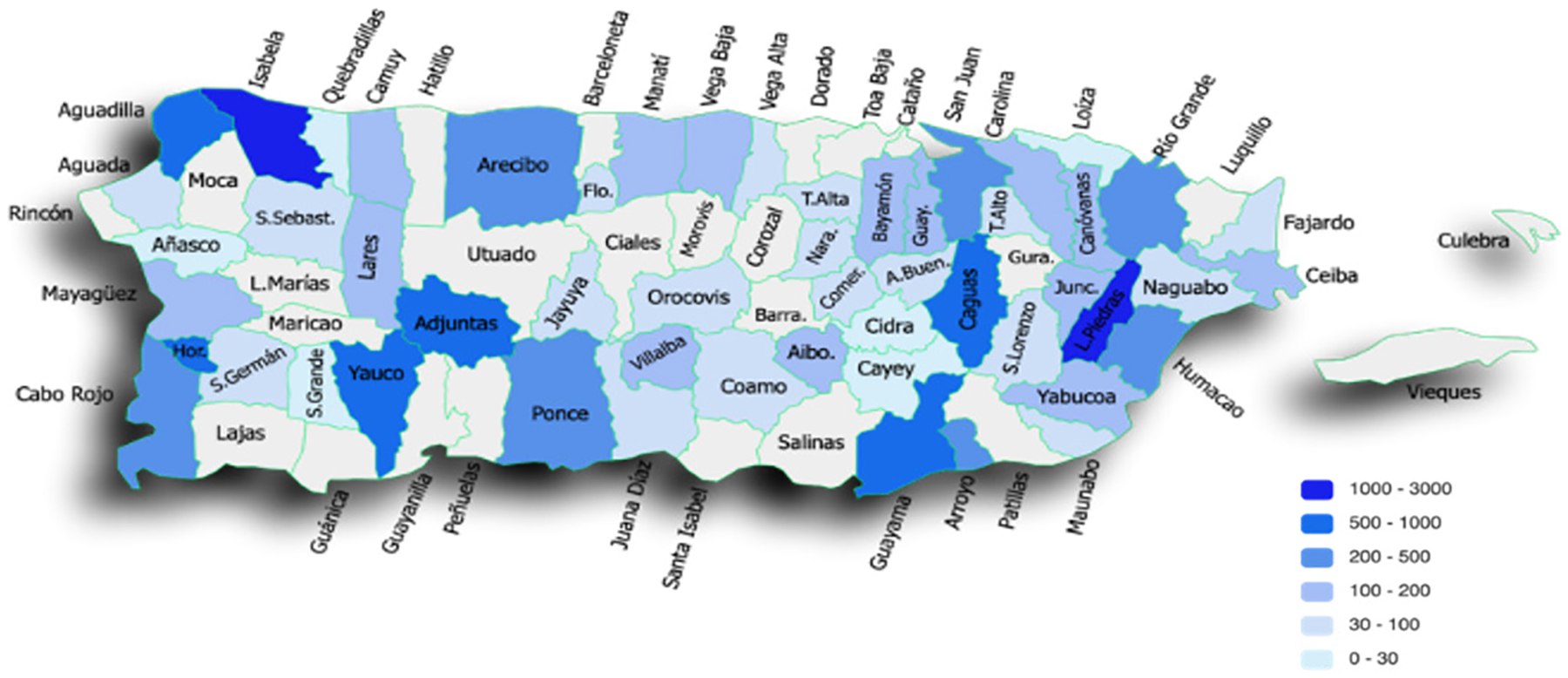
Number of people reached through participants’ STEM Ambassadors projects across Puerto Rican municipalities.

As part of the program, girls were encouraged, guided and supported to design and complete a STEM Ambassador outreach activity. To assess the impact of the STEM Ambassadors project, girls had to submit an evidence form that included a brief explanation of their activity and experience, the number of people reached, and videos or pictures of their activity. The STEM Ambassadors projects during the 2020 and 2021 cohorts reached an estimated 27,904 people from 54 municipalities across PR (Figure 3). Projects included a school group that created a filter to recycle collected rainwater, a school interactive science challenge for participants to learn basic STEM concepts, a public seminar on the impact of pollution in an afro-Puerto Rican community, social media campaigns, coding hours, and much more (see Appendix Table 1 for a sample of projects during the 2020 and 2021 cohorts). Each year, the impact of the STEM Ambassadors’ projects was celebrated through a press release, social media posts, and TV and radio interviews. Also, all Ambassadors’ projects descriptions and impact were shared on the CienciaPR webpage. The most creative and impactful STEM Ambassadors projects were recognized with STEM kit prizes (2020) or cash prizes (2021) during the closing ceremony. The winners were selected by an external committee of Seeds of Success sponsors and mentors who evaluated project reports delivered on time based on their innovation and relevance to STEM, as well as the number of people impacted.

### Mentors.

A total of 180 women in STEM (120 in 2020 and 60 in 2021) were recruited through the CienciaPR network (Guerrero-Medina, 2013) to mentor and advise program participants. Mentors had to self-identify as women, be highly proficient in Spanish, and either have an advanced degree in a STEM-related field or be in the final stages of a STEM bachelor’s degree and have significant research or engineering prior experience. Expectations for the mentoring relationship were communicated to students and mentors through a mentoring guide and reinforced during their respective Orientation Session. Mentors also received a brief mentoring training during the orientation that included effective strategies, including discussions of promoting a growth mindset, the importance of normalizing effort and failure and of sharing aspects of their identities the girls could relate with, and cultural awareness and sensitivity skills, as well as what to do if any problems arose or if the mentors were concerned about the girls’ safety. The program organized four online group meetings between mentors and students and mentors were also expected to talk with their mentee (by phone or video call) individually in the presence of a parent. Mentors were instructed to cover three main topics: (1) the girl’s STEM interests; (2) the mentor’s STEM trajectory, career, and advice; and (3) the girl’s STEM Ambassadors project.

In their post-intervention survey, most mentors (97.6%, combined 2020 and 2021) indicated that they had participated because they were interested in contributing to the development of girls in STEM disciplines and this was the goal that was most commonly accomplished at the end of the program (86.5%). A majority of mentors also agreed or highly agreed that the Seeds of Success program helped them feel confident being a role model for girls interested in STEM (84.8%), improving their mentoring skills for middle school girls (87.3%), contributing to the development of girls in STEM disciplines (92.4%), helping girls appreciate the importance of STEM disciplines for society (92.4%), inspiring girls to consider STEM careers (88.6%), and feeling motivated to seek other mentoring opportunities (90%). Overall, mentors greatly enjoyed their mentoring experience (mean of 9.5 out of 10, 10 being the highest). Here are some of the mentor’s testimonies:

“The most gratifying experience was to orient my girl about STEM careers, and through her, reach other girls…”

“The most gratifying experience was being able to share and let her know the opportunities in STEM that students can have. To ensure that girls are aware of the opportunities that exist, preventing them from leaving high school without guidance.”

Mentors were essential in guiding participants towards the completion of their STEM Ambassadors projects and providing constructive and helpful feedback in designing their projects, as pointed out by participants with a median value of 5 (strongly agree) for all satisfaction items related to mentoring (Table 2).

### Study Participants.

Before starting, participants and their parents were invited to take part in a study to assess the impact of the program. A total of 192 participants (79%) enrolled in the study and from this study sample, 127 (66%) completed all surveys. The school level and school type of enrolled study participants was comparable to program participants. Enrolled study participants were in the 7th (15%), 8th (39%) to 9th (46%) grades and came from public schools (63%), private schools (35%), and homeschooling (2%).

Based on our Pre-Intervention survey, 25% of study participants had previously taken STEM honors or advanced classes and only 14% had been enrolled in a special science or engineering program. Based on PR census data (US Census, 2022), most participants are considered Hispanic and are therefore part of an underrepresented group in science (NIH, 2019).

### Satisfaction.

According to the data reported in the Mid-Intervention and Post-Intervention Surveys, study participants were highly satisfied with the activities carried out. All survey items about program activity satisfaction received a median value of 4 or higher in a scale of 1–5, with 5 being the most positive score. Mean score across all activities for the 2020 cycle was 4.37 and 4.5 for the 2021 cycle. A total of 97% of the students “liked” or “really liked” the program. The activities that participants liked the most according to their answers in open-text survey questions were the workshops and getting to know STEM professionals.

### Reliability and Validity Testing of the Survey.

Mean values for item answers in the Pre/Post-Intervention Surveys ranged from 2.33 to 4.55. Skewness (range: −2.291, 0.477) and Kurtosis values (range: −0.986, 6.883) are acceptable to prove normal univariate distribution. KMO value of 0.744 and Barlett’s test (2330.265) and significance (<0.001) have shown to be adequate. Cronbach’s alpha value at 0.908 estimates a high internal consistency.

Exploratory Factor Analysis (EFA) with principal axis extraction method was used with an oblimin rotation method. A 6-factor solution was extracted that explained 47.25% of the variance (Table 3). For simplicity, we have labeled these factors as (1) Science Identity, (2) Program Objectives, (3) STEM Interest, (4) Leadership Identity, (5) STEM Attitudes, (6) Numerical Skills and Perception.

### Program Impact.

A Wilcoxon signed ranks test was used to compare the Pre- and Post-Intervention Survey scores for each individual item, for the six factors, (referred to here as categories), and for the survey as a whole. The categories of Science Identity, Program Objectives, STEM Attitudes, and Numerical Skills and Perception showed a significant difference, as seen in Table 4. STEM Interest and Leadership Identity did not increase significantly.

Most of the items in the Program Objective category showed a significant difference, revealing an increase in self-perception as a leader, confidence in their ability to succeed in science, knowledge about STEM careers and opportunities, and access to STEM role models after program participation (Appendix Table 2). In addition, PRE/POST surveys indicated an increase in the participants’ ability to identify Puerto Rican STEM professionals (p-value <0.001) and their ability to identify women STEM professionals (p-value <0.016). Overall, scores for the entire survey were higher after the program than before, as seen in Table 5. Moreover, 95% of participants declared the intention of participating in other STEM activities in the future, such as advanced STEM classes, STEM college studies, and/or science fairs after program participation.

### Participant Voices.

According to open-text answers in the Post-Intervention Survey, the program helped participants understand different branches of science, think about their professional goals, confirm their interests in STEM, and feel closer to achieving their professional goals.

“…Now I know more careers in STEM and I already have a clearer idea of what I want to study in the future.”

“The Seeds of Success program was an unforgettable experience. Thanks to the program I found a profession that interests me and that I hope to study in the future. I think it’s a very inspiring initiative, and personally seeing how participating in the program paid off is very gratifying.”

“I will always remember Seeds of Success as the program that began to open doors for me in order to be closer to fulfilling my dream of becoming a professional.”

Girls also expressed how meeting female STEM professionals and other girls with the same interests helped them realize the importance of STEM professions. Some stressed the importance of meeting a variety of STEM professionals.

“The thing that I most liked was being able to get to know different types of STEM jobs and realizing the importance of STEM professions.”

“I liked that there were many girls like me interested in learning.”

“What I liked the most was the interaction with my mentor. It taught me a lot and motivated me to become a scientist in the future. Her research area was very interesting.”

“I loved the mentoring. Having a mentor to help me organize my ideas was an experience I had never had. I really thank my mentor for helping me follow my dreams of being able to lead and present issues to my community with pride and confidence in myself.”

Interestingly, the “Juntas Podemos” webinars, helped participants see themselves as scientists, dismantle common science stereotypes, and realize that science and engineering are not innate abilities but rather skills you can develop.

“What I liked the most about Seeds of Success was the series of talks by the scientists, since it showed me that I too have the possibility of one day being one of them and fulfilling my dreams.”

“The thing I most liked about the Seeds of Success program was the “Juntas Podemos” series. I love these types of talks because they show you that scientists and engineers are ordinary people. You don’t have to be a genius to be a scientist. You just have to put your mind to it and work hard smartly.”

“What I liked the most were the “Juntas Podemos” talks, because I was able to hear how each of these women achieved what they wanted and never gave up.”

Participants also let us know that the STEM Ambassador projects helped them develop leadership skills. Specifically, they talked about how the project helped them develop perseverance, challenged them to implement new STEM initiatives in their communities, and made them feel good about sharing their knowledge with schoolmates.

“It helped me a lot to develop as a leader. It helped me feel that if I try hard enough I can be what I want.”

“The program helped me grow as a person. I have learned many things from different aspects of science. I even learned how to develop leadership and other important qualities. I am delighted to be part of a program like this.”

“What I liked most about Seeds of Success was developing my leadership skills through a science project and being able to share it with my schoolmates.”

“What I liked is that it is a different program that challenges you to do new things that you never thought would happen.”

“This helped me to be a leader, thanks to my work the school director allowed me to have a club at school. It was simple but the next school year we are designing it to be very motivational and very professional.”

## DISCUSSION

This article presents an example of a program designed to promote STEM interest among girls from historically underrepresented backgrounds by leveraging three strategies: (a) exposure to STEM experiences, (b) relatable role models, and (c) empowering place-based STEM outreach projects. Here we discuss details about the program’s implementation, evidence of short-term impact, and key programmatic components.

### Proactive Efforts for Broad and Diverse Recruitment.

Over the years we have developed a diversified set of strategies to ensure broad and diverse recruitment of girls throughout PR. Our program is benefitted by CienciaPR’s extensive community, popular website (the top science website for audiences from PR according to Google analytics), and longstanding and active presence on social media (Facebook, Instagram, Twitter, and LinkedIn accounts reaching >30K people per week). We have also established a collaboration with the DEPR to disseminate information about the program. Perhaps the factor that has helped most in ensuring broad visibility has been CienciaPR’s healthy relationship with media outlets and the use of a media relations consultant to issue press releases during recruitment periods and assure radio and TV coverage for past program participants and their STEM Ambassadors projects. We have also begun piloting novel social media recruitment strategies suggested and managed by past participants. For example, for the recruitment of the 2020 cohort, we partnered with Latinas in STEAM, an Instagram and Facebook movement established by a Seeds of Success participant as part of her STEM Ambassadors project. Involving past participants is aligned with one of our core beliefs that “if you can see it, you can be it.”

Our application evaluation rubric has allowed us to recruit a diverse set of participants with respect to school and geographic diversity and to target girls from underserved backgrounds as determined by past STEM experiences and school type (64% public school). Although we do not have data on girls’ race/ethnicity and family socioeconomic background (we have started collecting data with the 2022 cohort), we assume based on the most recent census that the majority of girls identify as Hispanic (98.8% of PR identifies as such, US Census Bureau, 2022). Similarly, though we did not collect socioeconomic data in 2020 or 2021, a majority of public school students in PR are from low socioeconomic settings (84%, DEPR, 2022) and in general 57% of children in PR live in poverty (Annie E. Casey Foundation, 2022). We thus estimate that a majority of the girls served come from low socioeconomic backgrounds. Going forward, we have identified the need to increase the number of applications from rural communities, particularly from municipalities that have not yet been part of our program, as well as from girls with disabilities. To do this we will be visiting rural school districts during teacher conferences to promote identification of students from these communities and adding resources to support the accessibility of both online and in-person activities.

### Ensuring Program Retention.

A majority of girls (64%) completed all components of the program in a synchronic fashion, including the STEM Ambassadors project. Given the relatively high number of activities, the length of the program, and the challenges of implementing a STEM Ambassadors project, this participation rate is to be expected. Importantly, a majority of girls (80%) participated in the mentoring sessions and remained engaged throughout the program, receiving a certificate of participation at the end (71%).

Over the years we have experimented with several strategies to promote participation, retention, and STEM Ambassadors project completion. A common cause for attrition encountered during discussions with the girls and their parents is a high load of classes at school. To counteract this effect, girls who are unable to participate live are given the opportunity to watch recordings of the workshops and “Juntas Podemos” webinars asynchronously so they may complete those requirements. We have also recently implemented an obligatory pre-application information session for girls and parents where we discuss required program activities and dates. Girls can choose to not finish the application process if they don’t think they will be able to participate in scheduled activities. In addition, we have implemented a time management panel featuring past program participants who share how they were able to balance school, personal activities, and the program activities. After implementing these strategies, we have seen an increase in girls’ retention.

We have also implemented a number of strategies to improve program communications that have been well-received. In the past we found that solely focusing on website content and email communication through the parents was not effective. Communications improved when we began developing dynamic media content (e.g., videos, gifs, graphics) and complementing emails with “Whatsapp” to send reminders, program communications, and celebrations of activity completion. We also developed a more attractive and defined branding and visual language that leveraged images and stories from past alumni and participating mentors. These were incorporated into program materials, including a printed calendar with important program dates. We believe this communication strategy has been critical for engaging and retaining girls throughout the program.

Finally, with respect to the STEM Ambassadors projects, we recognize that this is perhaps the most challenging aspect of our program. To facilitate completion and development of leadership skills, mentors are instructed to discuss and advise the girls about their projects, we provide a guide with examples of different types of projects they may want to undertake, we model the development of an STEM Ambassadors project idea through a design thinking exercise during the initial meeting, we secure collaborations with STEM or other community organizations in Puerto Rico that the girls could work with, we provide ongoing staff support, and we award a modest stipend ($50) to help with incidental expenses. In addition, we have begun to incentivize completion by awarding sponsor-donated prizes to the most creative and impactful projects per region during the closing ceremony when all girls present their final projects, and by highlighting as many projects as possible in our social media and in the media campaigns for the recruitment of the next cohort.

### Seeds of Success Improves Girls’ Attitudes Towards STEM and Science Identity.

Participants from the 2020 and 2021 cohorts indicated high levels of satisfaction with the program in general and with each of the activities in which they participated. A statistical analysis of survey responses before and after program participation demonstrated significant changes in participants’ responses to the survey as a whole, as well as with respect to grouped items related to their attitudes towards STEM, scientific identity, and confidence in numerical skills. A set of questions meant to examine achievement of the stated program objectives was also highly significant, indicating that the program increased girls’ knowledge about STEM careers and STEM opportunities, access to role models and mentors, and self-efficacy in the sciences. We suspect that a lack of change in the grouped items related to STEM Leadership and STEM Interest could be due to a high baseline given the fact that girls were selected based on their high interest in STEM and demonstrated leadership qualities. It could also be due to not having provoked changes in these areas or not having well-elaborated survey items to measure these changes. Interestingly, although the questions for Leadership Identity were not significant at the group level, an item from the program objectives examining girls’ confidence in their capacity to be a leader was highly significant. Similarly, a significant number of girls’ responses to the open-text questions referenced the development of leadership skills and a new self-regard as leaders.

Because our evaluation did not include a control group, we are unable to conclude that observed changes were caused by our program. However, studies have found that STEM interest and science identity tend to decrease among girls during middle school (McQuillan et. al., 2023), suggesting that our program countered prevalent trends. We are planning on recruiting comparator groups for future cohorts to examine the question of causality. We are also continuing to measure Seeds of Success alumni’s STEM interests, involvement in STEM activities and STEM Ambassador projects, plans to go to college, and enrollment in STEM majors through a longitudinal reflective diary, which will allow us to evaluate the persistence of changes and ultimate outcomes.

The results regarding increases in science identity are encouraging. High levels of science identity (i.e., envisioning oneself as a “science person”) have been shown to be related to persistence in STEM academic and career paths (Andersen and Ward, 2014; Dou et al, 2019). Science identity tends to vary by race/ethnicity in middle school and high school, with males and White students exhibiting higher science identity than girls or Black or Hispanic students (Andersen and Ward, 2014). Developing a strong science identity is especially critical and predictive of persistence for girls and students of color (Andersen and Ward, 2014; Vincent-Ruz and Schunn, 2018). Longitudinal studies will examine whether observed changes in science identity persist and result in STEM trajectories for our students.

### The Importance of Latina STEM Role Models.

Stereotypes that associate a male gender or majority-group identity with STEM disciplines can discourage girls or students from non-majority groups from considering STEM majors and careers (Starr and Leaper, 2019; Shapiro and Williams, 2012). A lack of diverse STEM role models in the face of negative STEM stereotypes may explain differences in science identity by gender (Kim et al., 2018). In PR in particular, there is a dearth of culturally-relevant educational resources in the Department of Education curriculum, including those that present Puerto Rican role models of either gender (González-Espada et al., 2015). Through Seeds of Success, we sought to provide girls with culturally-relevant female STEM role models through group and individual mentoring interactions, as well as through participation in the Juntas Podemos webinar series. To do this, we leveraged the CienciaPR platform, an established online network that serves “anyone with an interest in STEM and PR”. CienciaPR was built to counteract the negative effects of under-representation, marginalization, and geographic dispersion of Puerto Ricans in STEM by: 1) promoting scholarly and mentoring interactions among self-identified members of an otherwise dispersed community; 2) providing visibility to diverse scientific role models; and 3) connecting STEM professionals with educators, students, and media outlets to promote culturally-relevant formal and informal STEM education (Guerrero-Medina et al., 2013). Currently, the CienciaPR network has >16,000 members, 62% of whom identify as female, and who represent a broad geographic footprint (83% in PR and 17% in the US), wide range of disciplines (45% biological sciences, 10% health, 10% engineering or math, 9% physical or chemical sciences, etc), and a continuum across STEM training academic and professional stages (40% undergraduate, 12% graduate, and 39% STEM professionals). This network has been a great resource to many initiatives aimed at expanding the diversity of STEM experts, role models, mentors, science communicators, and students (Guerrero-Medina et al., 2013).

Quantitative results suggest that we were successful in exposing girls to relatable STEM role models. Similarly, it was encouraging to see mentoring relationships mentioned many times in the girls’ open-text responses regarding the aspects of the program that they most enjoyed. In-group role models can mitigate the negative effects of STEM stereotypes (Lawner et al, 2019) and boost STEM self-efficacy and identity (Stout et al., 2011). STEM programs that emphasize the congruence of science identity with girls’ individual identities increase positive attitudes towards STEM and motivation to persist in STEM, especially for girls from underrepresented backgrounds (Andersen and Ward, 2014; Oyserman and Destin, 2010; Diekman and Steinberg, 2013). We expect that the girls’ mentoring interactions and exposure to role models contributed to the observed increases in positive attitudes towards STEM as well as science identity. Results also validated our online mentoring approach, which we implemented because of COVID-19 but have since decided to keep due to the ease of participation for Latina STEM mentors, regardless of where they are located. Other programs have found similar results (Stoeger et al., 2013). Programs wishing to establish a mentoring component, particularly with populations that are underrepresented in STEM, may seek to replicate our approach by considering online interactions and partnering with national and international networks of scientists from minoritized backgrounds.

### The Impact of STEM Ambassadors Projects.

Seeds of Success provides girls the opportunity to form bridges between their social and scientific identities by guiding and supporting them to develop community-focused STEM Ambassadors projects of their own design. Community-relevant projects are an opportunity to engage students who do not see science as part of their cultural or social identity, or who perceive that STEM careers do not offer an opportunity to connect with others in their community (Fuesting and Diekman, 2017; Calabrese-Barton and Tan, 2010; Masnick et al., 2009). Presenting STEM topics and concepts that are connected to cultural, societal and community relevance is an effective strategy for promoting inclusive learning among middle school students (Vogt et al., 2016; Christy, 2016).

In addition to fostering the inclusion of different STEM learners, the girls’ STEM Ambassadors projects are intended to build agency and to demonstrate to girls that they possess the creativity and internal resources to complete a project and be change-makers in their communities. Girls have continually impressed us with their creativity and ambition. Although diverse in terms of topic, target population, and mode of delivery, some trends can be observed among STEM Ambassadors projects. Many of the girls’ projects promote female STEM role models, deliver concepts or skills through workshops, promote respect for the environment, or expose students to the excitement of STEM (see Appendix Table 1 for a sample of STEM Ambassadors projects). The creative projects have also helped amplify the reach and impact of Seeds of Success. In 2020 and 2021, the girls’ STEM Ambassadors projects (n=150) reached >27,000 people throughout Puerto Rico or an average of >185 people reached per project, surpassing our communicated expectation for a minimum of 30 people impacted per project. These achievements merit recognition, and thus we take care to celebrate STEM Ambassadors as leaders through our social media channels, as well as through local and national media outlets. In doing so, we hope to communicate to our participants, as well as other young people watching, that “they are the leaders of today, not tomorrow.”

### COVID-19 and Online Programs.

The delivery of program components through an online format was precipitated by COVID-19. Although these changes were initially burdensome and we worried about accessibility and the ability to engage students through a virtual format, we found that online activities can still be effective and in some cases may offer advantages. Specifically, through an online format it was possible for us to schedule more points of interaction throughout the calendar year than is logistically feasible with in-person meetings. It has been observed that when STEM exposure programs are conducted in out-of-school settings, success requires sustaining interactions over time (Thiry et al., 2017). We found that going online helped us achieve sustained interactions, especially between the girls and their role model mentors. Also, because online activities were not restricted to location, we were able to expand the numbers of female STEM role models from underrepresented backgrounds recruited to the program (Stoeger et al., 2013). Lastly, girls in PR from rural and mountainous communities can be at a disadvantage by having to travel far for in-person activities. Overall, the addition of the online format helped expand applications and minimize scheduling and transportation burdens placed on the girls and their parents.

We also worried that lack of access to internet and computer services could be problematic. During the on-boarding process, we asked girls about their ability to access the internet or computer devices, and any technology challenges they might face. Fortunately, we have received only a handful of complaints or reports about technology challenges over the past three years. We have found that many online meeting platforms (e.g., Zoom) may be accessed via mobile devices, which are prevalent in Puerto Rico regardless of income level. In cases of technical difficulties, we approach our network of partners and funders to help provide assistance and/or equipment.

All in all, results from our program evaluation lend support to online STEM outreach programs being effective in increasing science identity and improving STEM attitudes. We have come back to in-person activities for our most recent cohort (2022–2023), but we have kept several of the online activities, such as some of the STEM workshops and the group and individual STEM mentoring sessions.

### Conclusion and Long-Term Vision.

Here we present a program aimed at exposing Puerto Rican girls to culturally-relevant STEM female role models, mentors, and activities that inspire them to see themselves in these pathways and careers. Novel components include group and individual online mentoring sessions with Latina STEM experts and the development of identity, confidence, and leadership skills through a STEM Ambassadors program. We observe more positive attitudes towards STEM, higher science identity, and increased knowledge about STEM careers and STEM opportunities among girls who have completed the program. In future iterations, we are planning to assess causality through a matched-control design. We are also currently evaluating alumni’s long-term involvement in STEM, both in and out of school, and ultimately, their trends in college enrollment in STEM majors.

The program has been highly popular in PR securing hundreds of applicants each year and assuring participation of girls from underserved schools thanks to our use of traditional and social media, our targeted branding and marketing, and on-the-ground collaborations with schools and education systems. Going forward, we are hoping to secure funding to increase the number of participants in PR, particularly from rural and mountainous regions with high child vulnerability indices (Center for Puerto Rican Studies, 2022). In addition, through a grant from the National Institutes of Health, we are currently in the process of implementing our first Seeds of Success program in New Haven, CT, a primarily Latinx and African American community in the U.S. We are hoping that this new Seeds of Success program outside of PR will demonstrate the exportability of our model for the benefit of more girls, particularly those from historically excluded populations in STEM.

## Supplementary Material

Appendix. Additional Tables

1

## Figures and Tables

**Table 1.** Program participation throughout activities offered in 2020 and 2021.

Activity	2020	2021
Participants enrolled in the program	121	120
Leadership workshop	121	120
Chemistry/biology workshop	88	78
Coding workshop	79	72
Juntas Podemos webinar series	79	93
Mentoring program	97	95
STEM Ambassadors project	82	68
Met certificate requirements	88	83
Estimated STEM Ambassadors project impact	18,127	9,777

**Table 2.** Median value for items related to mentors and mentoring sessions.

Items	2020	2021
I liked the assigned mentors	5	5
Mentors demonstrated STEM experience	5	5
The mentors provided constructive and helpful feedback in designing my STEM Ambassador Project	5	5
The mentors motivated me to improve my STEM Ambassador Project	5	5
The mentors were respectful	5	5
The mentors answered my questions	5	5
I think this activity should be offered for the next year of Semillas de Triunfo	5	5

**Table 3.** Exploratory factor analysis and total explained variance of questions included in the Pre- Post-Intervention Survey.

Items	Factor						Total Variance Explained
	1	2	3	4	5	6	
Se mucho de ciencia [I know a lot about science]	0.78						10.54 (24.51%)
Puedo ayudar a las personas cuando tienen dudas de ciencia [I can help people when they have science questions]	0.74						
Soy buena en ciencia [I am good at science]	0.66						
No puedo explicar bien lo que hace un cientifico [I can’t quite explain what a scientist does]	0.63						
Domino los temas de ciencia [I understand science topics]	0.59						
Aprendo fácilmente nuevos temas de ciencia [I learn new science topics easily]	0.58						
La ciencia es fácil para mi [Science is easy for me]	0.55						
Cuánta confianza tienes en tu habilidad de ayudar a tus amigos a entender la clase de ciencias [How confident are you in your ability to help your friends understand science class]	0.50						
Me gusta hablar de ciencias con otras personas [I like to talk about science with other people]	0.45						
Cuánta confianza tienes en tu habilidad de desarrollar y realizar un experimento científico [How confident are you in your ability to develop and conduct a science experiment]	0.42						
Conozco sobre diferentes carreras en STEM [I know about different careers in STEM]		0.79					2.92 (6.80%)
Conozco de oportunidades que existen para mi en STEM [I know of opportunities that exist for me in STEM]		0.74					
Tengo confianza en mi capacidad de tener éxito en las ciencias [I am confident in my ability to succeed in science]		0.62					
Tengo un gran interés por estudiar disciplinas STEM [I have a great interest in studying STEM disciplines]		0.58					
Siento que tengo acceso a modelos a seguir y mentoras en STEM [I feel I have access to role models and mentors in STEM]		0.5					
Tengo confianza en mi capacidad de ser una líder [I am confident in my ability to be a leader]		0.46					
Me gustaría tener una carrera relacionada a las ciencias o la ingeniería [I would like to have a career related to science or engineering]			0.91				2.30 (5.35%)
Quiero a estudiar ciencias o ingeniería en la universidad [I want to study science or engineering at university]			0.87				
No tengo planes de tener una carrera en ciencias o ingeniería [I have no plans to have a career in science or engineering]			0.73				
En el futuro planeo participar en un campamento de ciencia, informática, robótica, o matemáticas [In the future I plan to participate in a science, computer science, robotics, or math camp]			0.58				
En el futuro planeo matricularme en clases avanzadas o electivas de ciencia o matemáticas [In the future I plan to enroll in advanced or elective science or math classes]			0.47				
Me gusta aprender ciencias [I like learning science]			0.45				
Mis amigos me ven como una persona que es buena en ciencia [My friends see me as a person who is good at science.]			0.4				
Los científicos trabajan en las cosas que ayudan al mundo [Scientists work on things that help the world]			0.34				
Si veo un problema en mi comunidad puedo hacer algo para resolverlo [If I see a problem in my community I can do something to solve it]				0.68			1.77 (4.12%)
Puedo convencer a otras personas a que me ayuden en mis proyectos [I can convince other people to help me in my projects]				0.67			
Soy una líder [I am a leader]				0.63			
Trabajo bien con otros en equipo [I work well with others in a team]				0.59			
En el futuro planeo competir en una feria de ciencias o matemáticas [In the future I plan to compete in a science or math fair]				0.50			
Cuánta confianza tienes en tu habilidad de construir algo mecánico que funcione [How confident are you in your ability to build something mechanical that works]				0.43			
Muchos ingenieros tienen otros pasatiempos e intereses aparte de la ingenieria [Many engineers have hobbies and interests other than engineering.]					0.62		1.53 (3.55%)
Cualquier persona puede ser ingeniero si se esfuerza [Anyone can be an engineer if they put in the effort]					0.60		
Uno tiene que sacar todas “A” en la escuela y la universidad para ser ingeniero [One has to get all “A’s” in school and university to be an engineer]					0.41		
Es importante saber ciencia para conseguir un buen trabajo. [It is important to know science to get a good job.]					−0.38		
Muchos cientificos tienen otros pasatiempos e intereses aparte de la ciencia [Many scientists have hobbies and interests other than science.]					0.23		
Cuánta confianza tienes en tu habilidad de diseñar un “app” para que otras personas puedan usar [How confident are you in your ability to design an “app” so that other people can use it]						0.42	1.26 (2.92%)
No me gusta aprender matemáticas [I don’t like learning math]						−0.4	
La mayor parte de las personas deben saber algo de ciencia [Most people should know some science]						−0.4	
Tengo una buena idea de lo que hacen los ingenieros [I have a good idea of what engineers do]						0.35	
Cuánta confianza tienes en tu habilidad de aprender a resolver ecuaciones de matemáticas [How confident are you in your ability to learn to solve math equations]						−0.3	
Uno tiene que ser súper inteligente para ser científico [You have to be super smart to be a scientist]						0.26	
Los ingenieros trabajan principalmente en cosas que no tienen nada que ver conmigo [Engineers mostly work on things that have nothing to do with me]						−0.2	

**Table 4.** Wilcoxon signed ranks test P-values for exploratory factor analysis categories of Pre- Post-Intervention Survey. Questions related to Program Objectives were not included in the 2020 survey.

Category	2020	2021
1. Science Identity	0.045*	0.018*
2. Program Objectives	N/A	<0.0001*
3. STEM interest	0.774	0.853
4. Leadership Identity	0.233	0.398
5. STEM Attitudes	0.021*	<0.0001*
6. Numerical Skills and Perception	0.003*	<0.001*

**Table 5.** Overall scores for the Pre- and Post-Intervention Survey.

Cycle Year	Survey Data	Value
2020	Total pre median	144
	Total post median	148
	p-value (Wilcoxon test)	0.0254
2021	Total pre median	169
	Total post median	178
	p-value (Wilcoxon test)	0.0002

## ABBREVIATIONS

ACS: American Chemical Society
DEPR: Department of Education of Puerto Rico
EFA: Exploratory Factor Analysis
KMO: Kaiser-Meyer-Olkin
PR: Puerto Rico
SEPA: Science Education Partnership Award
SISE: Science Identity Survey
STEM: Science, Technology, Engineering, and Mathematics
UPR: University of Puerto Rico
