# Supplementary material for: Seeds of Success: Empowering Latina STEM Girl Ambassadors Through Role Models, Leadership, and STEM-Related Experiences
Source: J STEM Outreach. Author manuscript; Available in PMC 2023 Oct 26. (PMC10601408)
Supplement: Appendix. Additional Tables [file NIHMS1938520-supplement-Appendix__Additional_Tables.pdf]

Empowering Latina Girls in STEM - Hernández-Matías et al.

Appendix. Additional Tables

Table 1. *Sample of Seeds of Success STEM Ambassadors Projects carried out during the 2020-2021 and 2021-2022 cohorts. Descriptions come from the girls' final project reports.*

Title	Summary
Microplastics in the Water	Organized a talk about microplastics, where they are found, and how they affect humans.
Away Mosquitoes!	Distributed information sheets to the community on how to prevent mosquito-borne diseases and created an Instagram page with informative videos.
Providing Help through Science	Planned a beach cleaning day at Humacao beach with a group of girls. Gave a talk about beach restoration and the importance of keeping beaches clean.
How to Return to School in Person in Times of Coronavirus	Gave a presentation to kindergarten students on coronavirus prevention. Carried out an experiment to demonstrate the importance of hand-washing in preventing contagion. In addition, an interview was conducted with a doctor specialized in the subject.
Know More about STEM Professions	Create a web page about science and engineering.
We Need More Allies	Create a presentation about the importance of women in science.
Artist Cells	Create a YouTube channel about the importance of the arts in STEM careers and how both things are interconnected.
STEM	Organized a TikTok social network about science and experiment tutorials to increase interest in studying STEM.
Awareness	Create a coloring book for elementary school with simple explanations about science professions related to nature and emphasis on how both men and women can excel in STEM fields.
Fun Professions in STEM	Presented an online-talk about fun professions in STEM. Carried out an experiment on the properties of different materials.
People with Physical Disabilities	Create a social network to provide information and inspiration about people who had difficulties throughout their career due to lack of support or a disability but were able to achieve their goals.
The Branches of Sciences	Design of a word search and crossword puzzle games about STEM.
Solar Charger	Create a 5-volt solar cell phone charger.
STEM for All	Offer a talk about women in STEM, gender inequality, and the diversity of careers in STEM.
Marine Contamination	Presentation about awareness about marine pollution and endangered species.
Water for Life	Create a homemade filter to use rainwater.
Learn About STEM	Create a social network about the importance of STEM and its professionals, and access to STEM education.
Geological Hazards in Puerto Rico	Held a panel with two women geologists to explain earthquakes. Carried out a hands-on activity about a seismograph.
Sky is Not the Limit!	Developed a conference to raise awareness about opportunities in science and unmask the stereotype that science is an exclusive field for men or for people with exceptional IQ.
STEM Breakout Video Game	Create an escape room with science challenges so participants had fun while learning about STEM.

Table 2. *Wilcoxon signed ranks test P-values for each Pre/Post-Intervention Survey item. Asterisk indicates significance.*

Category	Item	2020	2021
Numerical Skills and Perception	Uno tiene que ser súper inteligente para ser un científico [You have to be super smart to be a scientist]	0.1635	0.0791
Numerical Skills and Perception	No me gusta aprender matemáticas [I don't like learning math]	0.2362	0.7582
Numerical Skills and Perception	Los ingenieros trabajan principalmente en cosas que no tienen nada que ver conmigo [Engineers mostly work on things that have nothing to do with me]	**<0.0001***	**<0.001***
Numerical Skills and Perception	La mayor parte de las personas deben saber algo de ciencia [Most people should know some science]	**0.0486***	0.8686
Numerical Skills and Perception	Cuánta confianza tienes en tu habilidad de diseñar un "app" que otras personas puedan usar [How confident are you in your ability to design an "app" so that other people can use it]	0.7199	0.1916
Numerical Skills and Perception	Cuánta confianza tienes en tu habilidad de aprender a resolver ecuaciones de matemáticas [How confident are you in your ability to learn to solve math equations]	0.5141	**0.0002***
STEM Attitudes	Es importante saber ciencia para conseguir un buen trabajo. [It is important to know science to get a good job.]	0.5012	0.3912
STEM Attitudes	Uno tiene que sacar todas "A" en la escuela y la universidad para ser ingeniero [One has to get all "A's" in school and university to be an engineer]	**0.0489***	0.8187
STEM Attitudes	Muchos ingenieros tienen otros pasatiempos e intereses aparte de la ingeniería [Many engineers have hobbies and interests other than engineering.]	0.096	**0.0118***
STEM Attitudes	Cualquier persona puede ser ingeniero si se esfuerza [Anyone can be an engineer if they put in the effort]	0.4171	0.6973
STEM Attitudes	Muchos científicos tienen otros pasatiempos e intereses aparte de la ciencia [Many scientists have hobbies and interests other than science.]	**0.0025***	**0.0102***
STEM Attitudes	Los científicos trabajan en las cosas que ayudan al mundo [Scientists work on things that help the world]	0.1702	0.0887
Leadership Identity	Cuánta confianza tienes en tu habilidad de construir algo mecánico que funcione [How confident are you in your ability to build something mechanical that works]	0.1379	0.694
Leadership Identity	Soy una líder [I am a leader]	>0.9999	0.6488
Leadership Identity	Si veo un problema en mi comunidad puedo hacer algo para resolverlo [If I see a problem in my community I can do something to solve it]	0.0983	0.0539
Leadership Identity	Puedo convencer a otras personas a que me ayuden en mis proyectos [I can convince other people to help me in my projects]	0.4579	0.2069
Leadership Identity	Trabajo bien con otros en equipo [I work well with others in a team]	0.2128	0.9013
Leadership Identity	En el futuro planeo competir en una feria de ciencia o de matemáticas [In the future I plan to compete in a science or math fair]	0.4842	0.8593
Program Objectives	Tengo confianza en mi capacidad de ser una líder [I am confident in my ability to be a leader]	.	**0.0311***
Program Objectives	Conozco sobre diferentes carreras en STEM [I know about different careers in STEM]	.	**0.001***
Program Objectives	Tengo confianza en mi capacidad de tener éxito en las ciencias [I am confident in my ability to succeed in science]	.	**0.0044***
Program Objectives	Conozco de oportunidades que existen para mi en STEM [I know of opportunities that exist for me in STEM]	.	**0.0001***
Program Objectives	Tengo un gran interés por estudiar disciplinas STEM [I have a great interest in studying STEM disciplines]	.	**0.7033**
Program Objectives	Siento que tengo acceso a modelos a seguir y mentoras en STEM [I feel I have access to role models and mentors in STEM]	.	**0.0001***
Science Identity	No puedo explicar bien lo que hace un científico [I can't quite explain what a scientist does]	**<0.0001***	**0.0001***
Science Identity	Cuánta confianza tienes en tu habilidad de desarrollar y realizar un experimento científico [How confident are you in your ability to develop and conduct a science experiment]	0.1454	0.0771
Science Identity	Cuánta confianza tienes en tu habilidad de ayudar a tus amigos a entender la clase de ciencias [How confident are you in your ability to help your friends understand science class?]	0.6659	0.8626
Science Identity	Soy buena en ciencia [I am good at science]	0.7205	0.2079
Science Identity	Sé mucho de ciencia [I know a lot about science]	0.7205	0.3964
Science Identity	La ciencia es fácil para mí [Science is easy for me]	0.5412	0.3897
Science Identity	Me gusta hablar de ciencias con otras personas [I like to talk about science with other people]	0.1065	0.0513
Science Identity	Domino los temas de ciencia [I understand science topics]	0.1458	0.0579

Table 2 *(continued)*.

Category	Item	2020	2021
Science Identity	Aprendo fácilmente nuevos temas de ciencia [I learn new science topics easily]	0.3683	0.9275
Science Identity	Puedo ayudar a las personas cuando tienen dudas de ciencia [I can help people when they have science questions]	0.6245	0.2093
STEM interest	En el futuro planeo participar en un campamento de ciencia, informática, robótica, o matemáticas [In the future I plan to participate in a science, computer science, robotics, or math camp]	0.1978	0.7155
STEM interest	Mis amigos me ven como una persona que es buena en ciencia [My friends see me as a person who is good at science.]	0.8969	0.1366
STEM interest	Me gustaría tener una carrera relacionada a las ciencias o la ingeniería [I would like to have a career related to science or engineering]	0.1379	0.9817
STEM interest	Me gusta aprender ciencias [I like learning science]	**0.0396**[*]	0.1218
STEM interest	Quiero a estudiar ciencias o ingeniería en la universidad [I want to study science or engineering at university]	0.9795	0.7689
STEM interest	No tengo planes de tener una carrera en ciencias o ingeniería [I have no plans to have a career in science or engineering]	0.9082	0.3299
STEM interest	En el futuro planeo matricularme en clases avanzadas o electivas de ciencia o matemáticas [In the future I plan to enroll in advanced or elective science or math classes]	0.3507	0.4508
