# Supplementary material for: Seeds of Success: Empowering Latina STEM Girl Ambassadors Through Role Models, Leadership, and STEM-Related Experiences
Source: J STEM Outreach. Author manuscript; Available in PMC 2023 Oct 26. (PMC10601408)
Supplement: 1 [file NIHMS1938520-supplement-1.pdf]

ASSOCIATED CONTENT

Supplemental material mentioned in this manuscript can be found uploaded to the same webpage as this the manuscript.
